# Morphological and Molecular Identification of *Physaloptera alata* (Nematoda: Spirurida) in a Booted Eagle (*Aquila pennata*) from Portugal

**DOI:** 10.3390/ani13101669

**Published:** 2023-05-17

**Authors:** Zaida Rentería-Solís, David W. Ramilo, Ronald Schmäschke, Sandra Gawlowska, Jorge Correia, Filipa Lopes, Luís Madeira de Carvalho, Luís Cardoso, Isabel Pereira da Fonseca

**Affiliations:** 1 Institute for Parasitology, Centre of Infection Medicine, Faculty of Veterinary Medicine, Leipzig University, An den Tierkliniken 35, 04103 Leipzig, Germany; zaida_melina.renteria_solis@vetmed.uni-leipzig.de (Z.R.-S.); stomoxys59@gmail.com (R.S.); gawlowska@vetmed.uni-leipzig.de (S.G.); 2 CIISA—Centre for Interdisciplinary Research in Animal Health, Faculty of Veterinary Medicine, University of Lisbon, Avenida da Universidade Técnica, 1300-477 Lisboa, Portugal; p6199@ulusofona.pt (D.W.R.); jcorreia@fmv.ulisboa.pt (J.C.); madeiradecarvalho@fmv.ulisboa.pt (L.M.d.C.); ifonseca@fmv.ulisboa.pt (I.P.d.F.); 3 Associate Laboratory for Animal and Veterinary Sciences (AL4AnimalS), Portugal; 4 Faculty of Veterinary Medicine, Lusófona University—Lisbon University Centre, 1749-024 Lisbon, Portugal; 5 CERAS—Centro de Estudos e Recuperação de Animais Selvagens, Quercus Associação Nacional de Conservação da Natureza, 6000-909 Castelo Branco, Portugal; anafilipa.sl@gmail.com; 6 Department of Veterinary Sciences, and Animal and Veterinary Research Centre (CECAV), University of Trás-os-Montes e Alto Douro (UTAD), 5000-801 Vila Real, Portugal

**Keywords:** *Aquila pennata*, booted eagle, nematode, *Physaloptera alata*, Portugal

## Abstract

**Simple Summary:**

Birds of prey are important predators and scavengers at the top of the food chain, but their parasite diversity has not been well studied. The aim of this study was to identify several nematode specimens found in the gizzard of a booted eagle (*Aquila pennata*) during necropsy. Following morphological and molecular analysis, they were identified as *Physaloptera alata*, a circumstance which represents the first report of this species in a booted eagle from Portugal. A new genetic sequence for this parasite is now available in GenBank for future research on birds of prey. This information is crucial for understanding the parasitological fauna of these birds in Portugal, as well as for wildlife rehabilitation centers, disease ecologists, and wildlife professionals to take appropriate measures in the event of parasitic disease.

**Abstract:**

*Physaloptera* spp. are parasitic nematodes that infect the gastrointestinal tracts of many carnivores and omnivores. Although they are distributed worldwide, *Physaloptera* spp. have not been studied in raptors in Portugal. In this study, we report *Physaloptera alata* in a booted eagle (*Aquila pennata*) in Portugal. Adult nematodes were discovered in the gizzard of a young booted eagle, and morphological features were consistent with those of the genus *Physaloptera*. DNA was extracted and a PCR assay performed to amplify a region of the 18S small subunit of the ribosomal RNA gene and the cytochrome c oxidase subunit I gene. The resulting PCR products were Sanger-sequenced, and comparison with the available sequences in the GenBank database confirmed the initial morphological classification as *Physaloptera* sp. Phylogenetic analysis clustered the sequence within the *Physaloptera* group. The presence of this parasite in raptors from Portugal is of particular importance to wildlife rehabilitation centers, disease ecologists, and wildlife professionals. Furthermore, we produced a new genetic sequence and have added it to the GenBank database of parasites in birds of prey.

## 1. Introduction

Nematode parasites belonging to the genus *Physaloptera* can infect a broad range of hosts. *Physaloptera* spp. are distributed worldwide and can be found in the gastrointestinal tract of many carnivores, including birds of prey [1,2,3,4]. In felines, *Physaloptera* spp. can cause inflammatory and degenerative pathological changes [5,6]. They can have a complex life cycle, with arthropods such as cockroaches and beetles acting as intermediate hosts [7]. Small vertebrates could play a role in the life cycle as paratenic hosts by ingestion of an infected arthropod, while predators are usually definitive hosts [6,8,9,10,11].

Birds of prey play an important role as top predators in their biotope ecology [12]. Because of this, changes in the fitness of the raptor population can have important consequences for their ecosystem [1]. However, studying the pathogens that affect these predators involves a large variety of challenges. Many species of birds of prey have large territories of difficult access, some of them are migratory, and many of them are under a protected status in their countries of origin [4,13]. For these reasons, parasite diversity in raptorial birds has not been studied in depth [4,13,14]. A few reports aimed at elucidating the parasitic species infecting raptors have been conducted [1,2,3,4,13,14,15]. This makes the confirmation of morphological identifications via phylogenetic studies difficult.

We report the presence of adult *Physaloptera alata* in a booted eagle (*Aquila pennata*) for the first time in Portugal. Additionally, we confirm the morphological identification of the parasite using phylogenetic analysis.

## 2. Materials and Methods

### 2.1. Case Report

In August 2018, a young booted eagle (*A. pennata*) was admitted to CERAS (Centro de Estudos e Recuperação de Animais Selvagens, Quercus, Associação Nacional de Conservação da Natureza (Center for Studies and Recovery of Wild Animals, Quercus National Association for Nature Conservation)), a wildlife rehabilitation center located in Castelo Blanco, eastern–central Portugal. The animal was originally found in the municipality of Abrantes, also in eastern–central Portugal. The eagle weighed 453 g and was in very poor condition (1 on a scale from 0 to 5). Additionally, the specimen presented traumatic injuries due to collision with an unknown object. These injuries consisted of dislocation of the left shoulder, in addition to fractures of the elbow joint and ankylosis. The eagle was euthanized due to poor condition, sustained trauma, and permanent loss of flight ability. During necropsy, 31 adult nematode-like worms were found in the gastrointestinal tract (gizzard). The worms were collected in water and preserved in 90% alcohol for further analysis. Histological observations were not part of this study.

### 2.2. Morphological Identification of Parasite Species

Nematodes were mounted in glycerol and observed under a light microscope, Olympus BX 50 (Olympus, Tokyo, Japan). Parasite genus identification was performed using several morphological keys [16,17,18].

### 2.3. Parasite DNA Extraction and Amplification of the 18S Small Subunit rRNA Gene and the Cytochrome C Oxidase Subunit I Gene

Genomic DNA (gDNA) was extracted and purified from the nematodes using the DNeasy Blood and Tissue Kit (Qiagen, Hilden, Germany) following the manufacturer’s instructions. For molecular identification of the parasite species, two genes were targeted: the 18S small subunit tRNA gene (SSU 18S rRNA), and the cytochrome c oxidase subunit I (*cox1*) gene. For the amplification of SSU 18S rRNA, the primers Nem_18SF (5′-CGCGAATRGCTCATTACAACAGC-3′) and Nem_18SR (5′-GGGCGGTATCTGATCGCC-3′) were used to amplify a ≈ 900-base-pair (bp) fragment according to Floyd et al. [19]. PCR reactions consisted of 2.5 µL of DreamTaq™ Green Buffer (10x; Thermo Fisher Scientific, Dreieich, Germany), 0.2 µM of each deoxynucleoside triphosphate (dnp), 0.5 µM of each primer, and 2 U of DreamTaq Polymerase (Thermo Fisher). Additionally, 3 µL of worm gDNA was used as the DNA template. Finally, nuclease-free, DNA-free water was added up to a final volume of 25 µL. PCR conditions were as follows: an initial denaturation cycle of 5 min at 94 °C followed by 35 cycles of denaturation at 94 °C for 30 s, annealing at 54 °C for 30 s, and extension at 72 for 1 min. A final extension cycle of 72 °C for 7 min was added.

Amplification of the *cox1* gene (≈650 bp) was performed using a primer cocktail as described by Prosser et al. [20]. PCR reactions and thermal cycling conditions were conducted exactly as described before [20]. gDNA from a previously identified Spirurida nematode (*Diplotriaena obtuse*) [21] was used as positive control for both PCR assays. Finally, all PCR reactions were run in a Biometra Tadvanced^®^ Thermal cycler (Analytik Jena, Jena, Germany) and visualized in a 1.5% agarose gel.

### 2.4. Cloning, Sequencing, and Phylogenetic Analysis

Fresh SSU 18S tRNA PCR products were cloned into a pCR™2.1 vector using the TA Cloning™ Kit (Invitrogen, Dreieich, Germany) following the kit’s instructions. Afterwards, plasmid DNA was prepared using the GeneJet Plasmid Miniprep Kit (Invitrogen, Dreieich, Germany) according to the instructions in the kit. Plasmid minipreps were commercially sequenced (Microsynth Seqlab, Göttingen, Germany) in both directions using the SSU 18S tRNA primers mentioned above.

*Cox1* amplicons were purified using the GeneJET PCR Purification kit (Thermo Scientific, Dreieich, Germany) and bidirectionally sequenced (Mycrosynth Seqlab, Göttingen, Germany) using the primers M13F (5′-TGTAAAACGACGGCCAGT-3′) and M13R (5′-CAGGAAACAGCTATGAC-3′) [22], as described by Prosser et al. [20].

Produced sequences were analyzed using the MEGA X software (version 10.1.5) [23] and blasted in the GenBank database. Annealing was conducted using the MUSCLE Software (https://drive5.com/muscle/ accessed on 12 May 2023) and evolutionary relationships between sequences were constructed using the maximum likelihood method and Kimura 2-parameter model [24]. Finally, uncorrected pairwise (*p*) distances between gene sequences were also calculated using the MEGA X software.

## 3. Results

### 3.1. Morphological Identification of Physaloptera *sp.*

**Figure 1:**
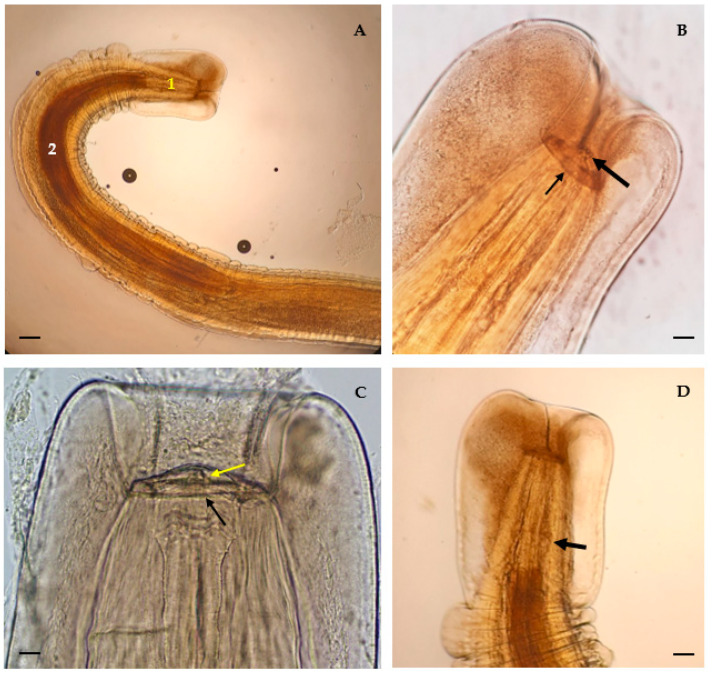
Morphological aspects of *Physaloptera* sp.: (**A**) anterior region of a female—muscular (1) and glandular (2) regions of esophagus (scale bar: 213.8 µm); (**B**) mouth opening of a female—cephalic collar (thin arrow) and teeth (thick arrow) (scale bar: 75.6 µm); (**C**) mouth opening of a male—cephalic collar (black arrow) and teeth (yellow arrow) (scale bar: 49.8 µm); (**D**) nerve ring (thick arrow) (scale bar: 106.9 µm).

The specimens had a thick cuticle and were striated transversely and detached from the body, with well-marked annulations (Figure 1A). At the anterior part, a cuticle dilatation was reflected over the base of the lips, forming a cephalic collar with small teeth (Figure 1B,C). The mouth had two large lateral lips (Figure 1B,C) with teeth on their internal surface and with papillae externally. The buccal cavity was short, and the esophagus was divided into two parts: a shorter muscular clear portion, and a longer glandular opaque portion (Figure 1A). The nerve ring encircled the esophagus in the beginning of its third posterior portion (Figure 1D). These anatomical characteristics allowed us to identify the specimens as belonging to the genus *Physaloptera*.

### 3.2. Morphological Identification of Physaloptera alata

**Figure 2:**
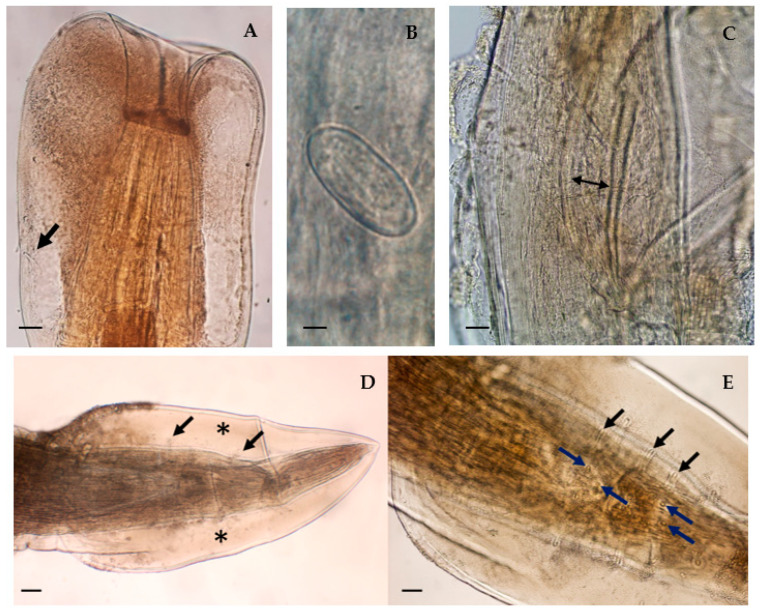
Morphological aspects of *Physaloptera alata*: (**A**) anterior end of a female specimen—deirid (thick arrow) (scale bar: 76.3 µm); (**B**) embryonated egg (scale bar: 9.7 µm); (**C**) male spicules (double arrow) (scale bar: 34.8 µm); (**D**) posterior end of an adult male, upon which large caudal alae (asterisk) and pedunculated papillae (black arrows) are visible (scale bar: 56.5 µm); (**E**) pedunculated (black arrows) and sessile (blue arrows) papillae are present anteriorly and posteriorly of the anus (scale bar: 141.2 µm).

Female: Total body length—24.5 mm; entire esophagus is approximately 1/5 of body length (17.4%) and tail is approximately 1/21 of body length (4.5%). Deirids located near the end of muscular esophagus (Figure 2A). Muscular esophagus 511 µm long, maximum width 128 µm; glandular esophagus measuring 4.3 mm long, maximum width 325 µm. Length ratio of muscular and glandular parts 1:8.4 (Figure 1A). Large anterior cuticular expansion, surpassing the anterior end and forming an invagination of the apical region (Figure 1B). Embryonated eggs with a thick shell, measuring, on average, 49.1 µm in length and 24.6 µm wide (Figure 2B).

Male: Total body length—21.7 mm; male spicules are equally sized (Figure 2C), measuring 275 µm. Caudal ala is large and very elongated, uniting anteriorly to the anus (Figure 2D). Posterior end with five pairs of pedunculated papillae and four pairs of sessile papillae (Figure 2E).

Comparing these morphological characteristics with those described by Cram [25] and Morgan [26], the specimens were identified as *P. alata*. Female and male gross specimens are deposited at CIISA, Faculty of Veterinary Medicine, University of Lisbon, under references “FMV-ULisboa-2023-01-Pa” and “FMV-ULisboa-2023-02-Pa”, respectively.

### 3.3. Molecular Identification of Physaloptera *spp.* and Phylogenetic Analysis

The presence of bands of specific lengths during agarose gel visualization confirmed the successful amplification of the *SSU 18S rRNA* and *cox1* genes. Sequences of the *SSU 18S rRNA* amplicon clones showed the highest identity percentage (99.31%) and a query cover of 100% when aligned to a *P. alata* 18S rRNA sequence (GenBank accession number: AY702703.1) with a query cover of 100%. This significant alignment was followed by partial sequences of the *SSU 18S rRNA* gene from *Physaloptera apivori* (98.85% identity, 100% query cover, accession number EU004817.1), and *Physaloptera* sp. (98.27% identity, 100% query cover, accession number: MG808040.1). The sequence produced in the present study was deposited in the GenBank database under accession number MN855524.

**Figure 3:**
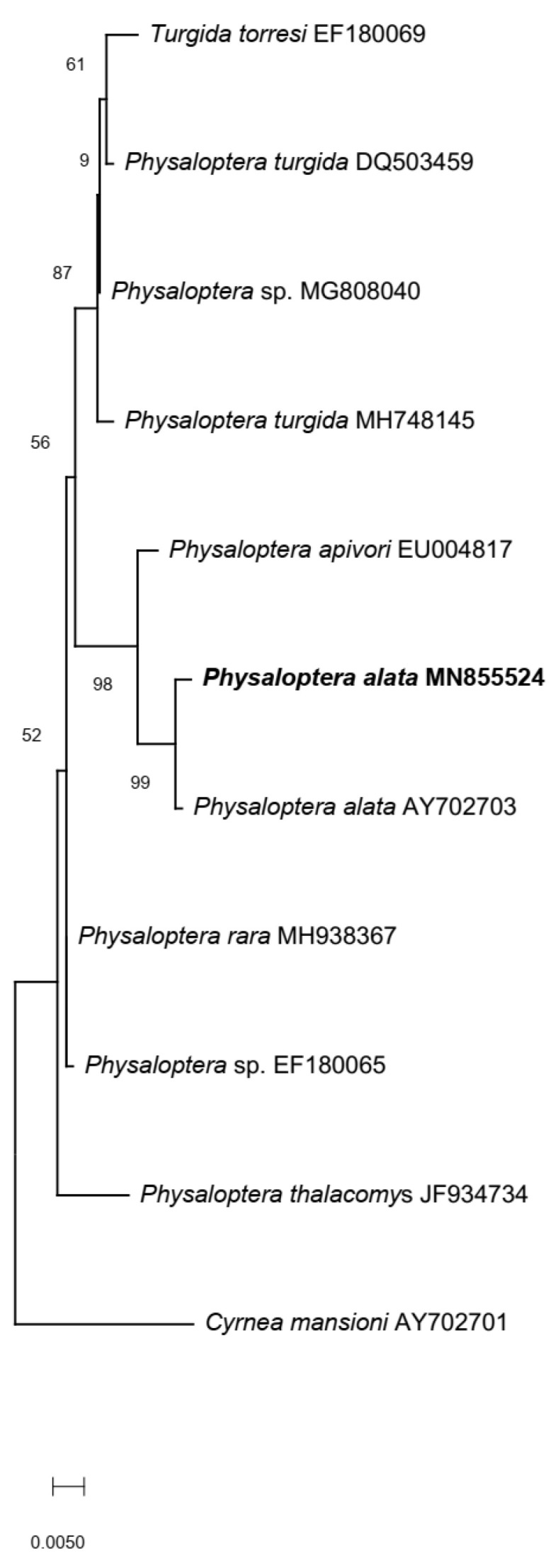
Phylogenetic tree of different nematodes of the order Spirurida from different vertebrates. The evolutionary distances between 11 nucleotide sequences of the SSU 18S rRNA gene were calculated using the maximum likelihood method and Kimura 2-parameter model. The sequence obtained in this report (in bold) is branched together with other *Physaloptera* spp. and clustered together with a sequence of the species *Physaloptera alata*. The tree is rooted with a *Cyrnea mansioni* sequence. Bootstrap values: 1000 replicates.

A dataset of 11 sequences of the *SSU 18S rRNA* gene, including the ones generated in this study, was used to build phylogenetic trees (Figure 3). The selected sequences belonged to other species of suborder Spirurina. Additionally, a *Cyrnea mansioni* sequence also from birds of prey was used to root the tree. Phylogenetic analysis clustered the sequence of this project within the genus *Physaloptera* (order Spirurida) and closely related to a *P. alata* (accession number: AY702703) sequence obtained from birds of prey in Germany [3] (Figure 3).

**Figure 4:**
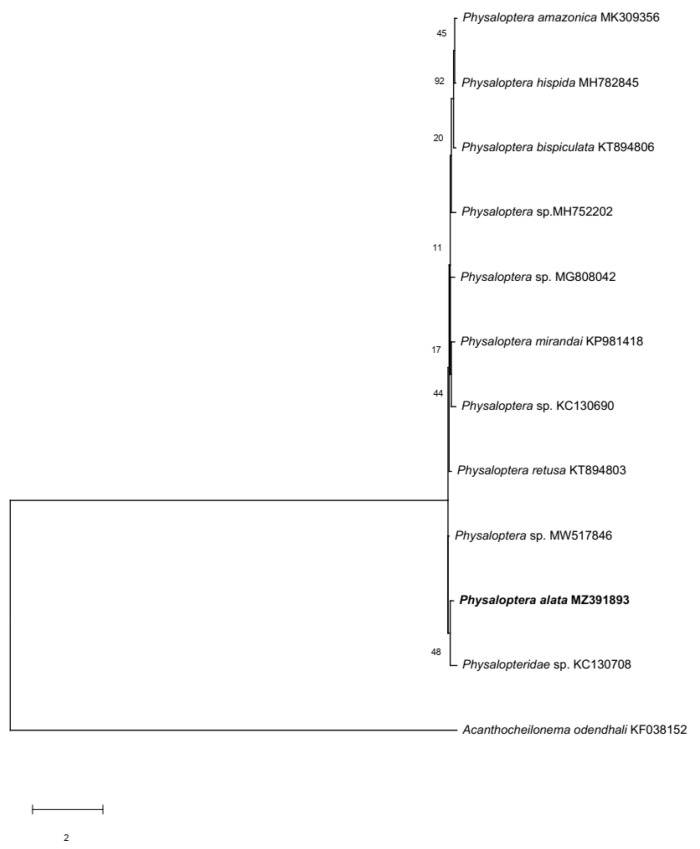
Phylogenetic tree of 12 *cox1* gene sequences. The *Physaloptera alata* sequence from this study (in bold) was also branched within the *Physaloptera* spp. group. An *Acanthocheilonema odendhali* sequence was used to root the tree. The evolutionary history was inferred with the maximum likelihood method and Kimura 2-parameter model. Bootstrap values for both trees: 1000 replicates.

Separately, the highest *cox1* gene identity percentage for our sequence was 85.07% with a sample from *Physaloptera* sp. (accession number MW5146) collected from a stray cat [27], followed by *Abbreviata caucasica* (syn. *Physaloptera mordens*) (accession number: MT23195) from a chimpanzee with 81.71% similarity [28]. After the estimation of evolutionary relationships, the *cox1* gene sequence from this study was found to be clustered in the *Physaloptera* spp. branch (Figure 4).

## 4. Discussion

We report *P. alata* in a booted eagle from Portugal. Booted eagles live in South Africa and migrate to the Iberian Peninsula and other Mediterranean regions to breed. Nevertheless, they are scarcely found in Europe [29]. A study found *P. alata* in one booted eagle from Spain in 1993 [1]. To our knowledge, this is the only European record that precedes this present report.

Besides booted eagle, *P. alata* has been recorded in peregrine falcons [3], Asiatic sparrowhawks (*Accipiler nisus*) [26], and sharp-skinned hawks (*Accipiter velox*) [26]. In Portugal, *P. alata* has been reported in birds of the orders Falconiformes and Strigiformes [30]. The present study is the first report in a Portuguese booted eagle.

Despite the available information, most of these previous studies were restricted to sole reports of parasite presence in a certain species, with small sample sizes [1,2,3,4,13,14,15,18,31,32]. In these studies, parasite species differentiation was based on morphological features only. Morphological traits are a pivotal and important tool for taxonomic classification; however, they can be difficult to recognize for non-experienced parasitologists.

The molecular identification of a determined species can help to confirm previous studies based on morphological features. In addition, they could also bring more information into the evolutionary relationships of different organisms. To our knowledge, only one study in nematodes from birds of prey has used genetic data to identify nematode species, including one *Physaloptera* sp. [3]. Currently, phylogenetic classifications of *Physaloptera* spp. in birds have been studied using the SSU 18S rRNA gene [3] and the *cox1* gene [20,30].

Honisch and Krone [3] used the SSU 18S rRNA gene to further study the phylogenetic relationships of Spirurina found in raptors; the authors produced SSU 18S rRNA partial sequences of one *Physaloptera* nematode: *P. apivori* (accession number: EU004817). Additionally, they used an unpublished sequence identified as *P. alata* (accession number: AY702703, found in a peregrine falcon) in their study.

During the phylogenetic analysis, the sequence generated in our investigation was found to be closely related to the *P. alata* sequence (AY702703). However, these data come from unpublished results and no morphological identification of the specimen was available. Additionally, not all of the *Physaloptera* spp. reported in birds of prey have been sequenced. This is why our combination of morphologic evaluation and phylogenetic analysis of a female and a male specimen was pivotal for the identification and confirmation of *P. alata* as the species found in this eagle.

To our knowledge, no *cox1* sequence from the *Physaloptera* worms of raptors was available for comparison. This only allowed the identification of the specimen to the genus level. A similar challenge was found by Kalyanasundaram et al. [33], who solely used the *cox1* gene to determine the species of a spirurid nematode detected on bobwhites. However, since there are limited resources in the gene database of Spirudia *cox1*, the authors could only identify the bobwhite parasite as *Physaloptera* sp. With this study, we provided the first *P. alata* partial sequence from raptors for the *cox1* gene, with the hope that future samples join ours and a larger database can be built.

Raptor research faces a considerable number of challenges, which makes conservation efforts, including health monitoring, difficult [34]. Given their pivotal role as top predators in the ecosystem, it is necessary to increase measures in order to elucidate raptor health. In the particular case of the booted eagle, although they migrate to southern Europe to breed each year, they are poorly studied and rarely found [29]. The lack of reports of parasites infecting these birds is most likely due to the scarcity of samples and specimens to study. Animals that are legally found and sent to rehabilitation centers, such as in this case, represent a unique opportunity to collect data about raptor health status and their parasitic fauna.

## 5. Conclusions

The present study is a small but necessary contribution to current efforts aimed at elucidating raptor health. Additionally, these results add information to the current database of *Physaloptera* spp. in these birds of prey. Finally, we encourage wildlife professionals, veterinarians, parasitologists, and ecologists to report and communicate similar cases.

## Data Availability

The data presented in this study are available within the article.
